# Circulatory context determines the anti-inflammatory efficacy of post-sepsis vagus nerve stimulation

**DOI:** 10.1016/j.jphyss.2026.100111

**Published:** 2026-10-15

**Authors:** Norito Washimine, Kaori Yagishita, Sayumi Matsuo, Kanoko Washimine, Chia-Hsien Wu, Yasuna Nakamura, Ryusuke Umene, Tomoya Nishino, Tsuyoshi Inoue

**Affiliations:** a Department of Physiology of Visceral Function and Body Fluid, Graduate School of Biomedical Sciences, Nagasaki University, Nagasaki, Japan; b Department of Nephrology, Graduate School of Biomedical Sciences, Nagasaki University, Nagasaki, Japan

**Keywords:** Cholinergic anti-inflammatory pathway, Vagus nerve stimulation, Sepsis, Lactate, Fluid therapy

## Abstract

Activation of the cholinergic anti-inflammatory pathway (CAP), including vagus nerve stimulation (VNS), suppresses inflammatory cytokine production and has shown efficacy in experimental sepsis when applied prior to sepsis induction. However, VNS before sepsis onset is rarely feasible in clinical practice, and whether VNS remains effective after sepsis onset is unclear. Using a lipopolysaccharide-induced mouse sepsis model, we evaluated VNS applied before and after sepsis onset. VNS applied before sepsis onset significantly reduced plasma TNF-α levels, whereas VNS applied after sepsis onset failed to suppress inflammation. Although post-sepsis VNS did not worsen hypotension or organ dysfunction, it was associated with increased lactate accumulation. Notably, fluid resuscitation restored the anti-inflammatory effect of VNS and reduced lactate levels. These findings suggest that CAP responsiveness depends on the host’s physiological state. Impaired circulation and metabolic stress may limit CAP activation during sepsis, while circulatory stabilization appears necessary for effective inflammatory control.

## Highlights

• Pre-sepsis VNS suppresses systemic inflammation in LPS-induced sepsis.
• Post-sepsis VNS fails to inhibit inflammation despite preserved blood pressure.
• Therapeutic VNS after sepsis increases lactate accumulation.
• Fluid therapy restores anti-inflammatory effects of post-sepsis VNS.
• Circulatory status determines efficacy of neuroimmune modulation in sepsis.

## Graphical Abstract

**Figure:**
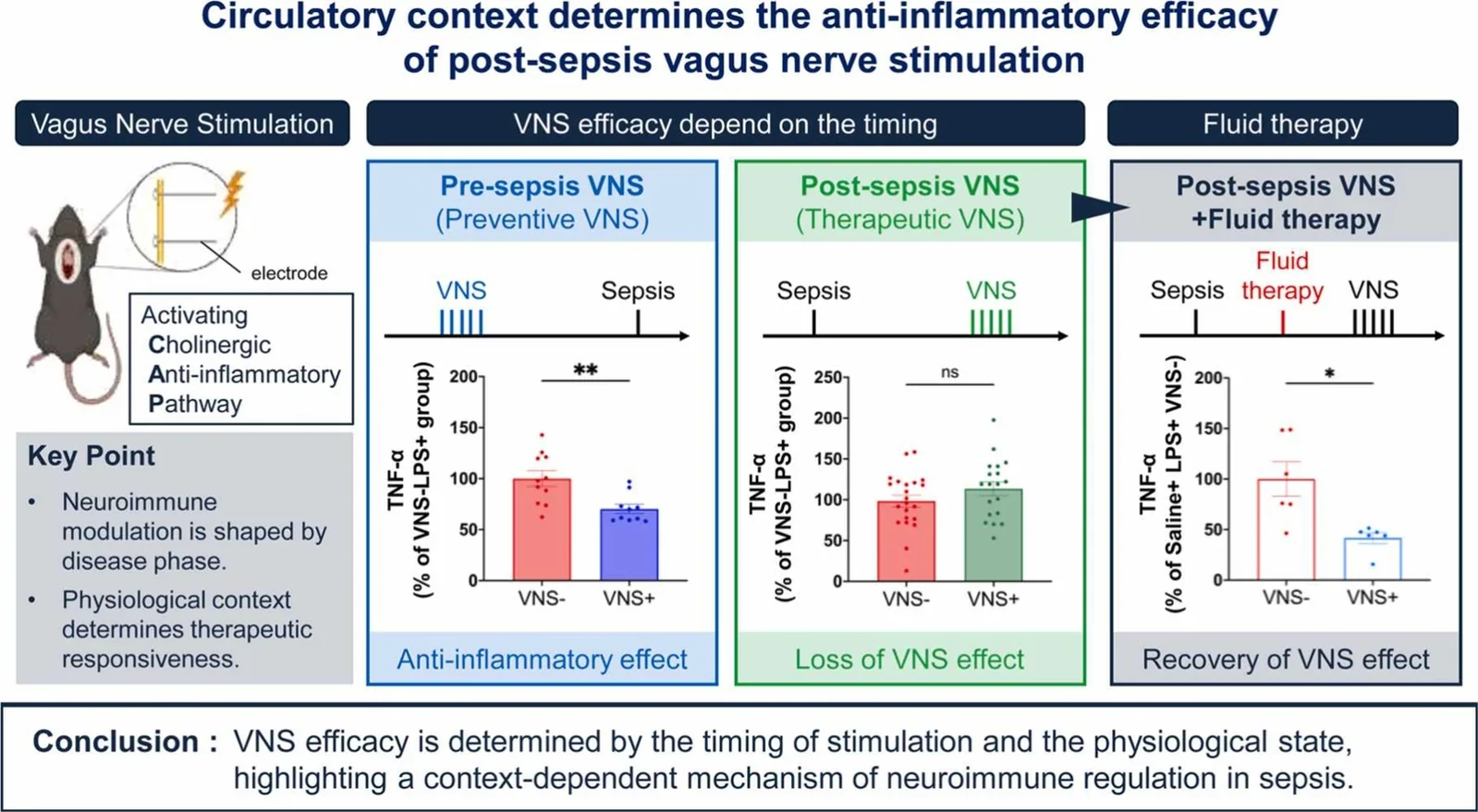

## Introduction

Sepsis is a life-threatening condition characterized by an excessive systemic inflammatory response that leads to multiple organ failure and high mortality. Despite advances in antimicrobial therapy and intensive care management, effective therapeutic strategies that directly target the inflammatory response itself have not yet been established. The fundamental pathophysiology of sepsis involves uncontrolled cytokine release and disruption of immune homeostasis, and elucidating the physiological mechanisms that regulate this response remains an important challenge.

In recent years, the cholinergic anti-inflammatory pathway (CAP) has been recognized as an important physiological mechanism through which the nervous system regulates inflammation. Borovikova et al. first demonstrated that vagus nerve stimulation (VNS) suppresses endotoxin-induced systemic inflammation [1], and Tracey subsequently conceptualized this mechanism as the “inflammatory reflex” [2]. CAP functions as a neural circuit from the central nervous system to peripheral organs. VNS activates choline acetyltransferase (ChAT)-positive β₂-adrenergic receptor–expressing T cells via the splenic nerve, and acetylcholine released from these T cells suppresses inflammatory cytokine production by macrophages through activation of the α7 nicotinic acetylcholine receptor (α7nAChR) [3], [4]. Downstream signaling mechanisms include inhibition of NF-κB nuclear translocation, activation of the JAK2/STAT3 pathway, and suppression of the NLRP3 inflammasome, resulting in reduced production of proinflammatory cytokines such as TNF-α [2].

Based on this mechanism, VNS has been proposed as a promising therapeutic target for chronic inflammatory diseases such as rheumatoid arthritis and inflammatory bowel disease. The development of transcutaneous vagus nerve stimulation devices has also facilitated clinical investigation of neuroimmune modulation [5]. Furthermore, recent studies have demonstrated that abdominal ultrasound stimulation can activate afferent vagus nerve fibers and induce anti-inflammatory effects through CAP [6]. The development of non-invasive technologies has paved the way for the clinical application of neuroimmune modulation via CAP activation.

Most previous studies investigating VNS in sepsis have utilized preventive intervention models in which stimulation is applied before the induction of inflammation. In clinical settings, intervention before sepsis onset is rarely feasible, and the therapeutic efficacy of VNS after disease onset therefore remains unclear. Recent studies, however, suggest that activation of the CAP may still exert protective effects even after tissue injury or systemic inflammation has already developed. In the field of acute kidney injury, VNS applied after the onset of cisplatin-induced nephropathy has been shown to confer renal protection [7]. In addition, the α7nAChR agonist GTS-21 reportedly attenuates inflammation even when administered after sepsis onset [8]. These findings raise the possibility that post-onset activation of CAP may retain therapeutic potential, although whether direct electrical stimulation of the vagus nerve itself is effective under septic conditions remains unknown. Sepsis is frequently accompanied by severe circulatory failure, tissue hypoperfusion, and lactic acidosis [9]. Additionally, adequate extracellular fluid resuscitation is recommended in the acute phase of sepsis treatment and exerts protective effects against circulatory failure and tissue hypoperfusion [10]. Since the CAP functions as a reflex arc, its activity may depend on systemic hemodynamic and metabolic conditions.

This study aimed to determine whether VNS maintains its anti-inflammatory effects after the onset of sepsis and to clarify how systemic circulatory status, particularly fluid therapy, influences the functional integrity of the neuro-immune reflex.

## Materials and methods

### Animals

Male C57BL/6 J mice (8–15 weeks of age) were used for all experiments. Mice were purchased from CLEA Japan Inc. (Tokyo, Japan) and housed at the Research Center for Biomedical Models and Animal Welfare, Graduate School of Biomedical Sciences, Nagasaki University. Animals were maintained under specific pathogen–free conditions in individually ventilated cages with controlled environmental conditions (temperature 23 ± 2 °C, humidity 50 ± 10%) and a 12-h light/dark cycle. Standard rodent chow and water were provided *ad libitum*. Only male mice were used in this study. All surgical procedures and euthanasia were performed under general anesthesia by intraperitoneal injection of a mixture of medetomidine (0.3 mg/kg), butorphanol (5 mg/kg), and midazolam (4 mg/kg), and blood samples were collected for further analyses. All animal experiments were approved by the Animal Care and Use Committee of Nagasaki University (Approval number 2503262030) and conducted in accordance with the Guidelines for the Care and Use of Laboratory Animals and the ARRIVE guidelines.

### Sepsis model

Experimental sepsis was induced by intraperitoneal injection of lipopolysaccharide (LPS) from *Escherichia coli* O111:B4 (L4391; Sigma-Aldrich) at a dose of 5 mg/kg. Control mice received an equal volume of sterile saline. Blood samples were collected at 5 h after experiment initiation to evaluate inflammatory responses and biochemical parameters. Plasma tumor necrosis factor-α (TNF-α) levels were measured as a marker of systemic inflammation. Blood cell counts, creatinine (Cr), blood urea nitrogen (BUN), and total bilirubin (T-Bil) were measured in blood samples. Acid–base status, including pH, bicarbonate, and pCO₂, and electrolyte concentrations were measured using a portable blood-gas analyzer (GASTAT-Pro; Techno Medica, Japan). The anion gap was calculated as follows: Na⁺ − (Cl⁻ + HCO₃⁻). Lactate concentrations were measured using a portable lactate analyzer (Lactate Pro 2, LT-1730; ARKRAY, Japan).

### Vagus nerve stimulation

VNS was performed based on a previously described protocol [3]. VNS was administered either 1 h before LPS injection (pre-sepsis VNS) or 1 h after LPS injection (post-sepsis VNS) under general anesthesia. A midline cervical incision was made, and the submandibular glands were retracted to expose the carotid artery and adjacent vagus nerve. The left cervical vagus nerve was then carefully isolated and placed on a bipolar silver-wire electrode. Electrical stimulation was applied using the following parameters: square-wave pulses at an intensity of 50 μA, a frequency of 5 Hz, and a pulse width of 1 ms for 10 min. In sham-operated animals, the vagus nerve was exposed but not stimulated. The left vagus nerve was covered with liquid paraffin to prevent drying before and during stimulation.

### Fluid resuscitation

For fluid resuscitation experiments, sterile saline was administered intraperitoneally at a volume of 20 μL/g body weight. The fluid volume was selected based on previously reported fluid resuscitation protocols in murine models of sepsis [11]. In the post-sepsis VNS experiments, saline was administered 30 min after LPS injection, followed by VNS or sham surgery 30 min later (1 h after LPS administration).

### Enzyme-linked immunosorbent assay (ELISA)

Plasma TNF-α levels were measured using a commercially available enzyme-linked immunosorbent assay (ELISA) kit (TNF alpha Mouse Uncoated ELISA Kit with Plates; 88–7324–88, Thermo Fisher Scientific) according to the manufacturer’s instructions. Blood samples were centrifuged at 7000 × g for 5 min at 4°C, and plasma was collected for analysis. Absorbance was measured using a plate reader.

### Blood pressure measurement

Blood pressure was measured noninvasively using a tail-cuff system (MK-2000ST NP-NIBP monitor, Muromachi Kikai Co., Ltd., Tokyo, Japan). Mice were acclimatized to the blood pressure measurement procedure for one week prior to experiments. In the post-sepsis VNS experiment, SBP was measured before LPS or vehicle administration and hourly thereafter until 5 h after administration. The percentage change in SBP was calculated as follows: [(SBP at 5 h − baseline SBP)/baseline SBP] × 100.

### Arterial line (A-line)

Mice were anesthetized with intraperitoneal anesthesia (as described for the VNS experiments) and, in the supine position, the median neck incision was made to expose the right carotid artery. After complete ligation of the cephalic side with 5–0 silk thread, an arterial catheter made of BD Intramedic PE Tubing (BD427401, Becton, Dickinson and Company, Franklin Lakes, NJ, USA) was inserted from its central side. It was connected to the Disposable BP Transducer (MLT0670, ADInstruments, Colorado Springs CO, USA), and arterial pressure was monitored over time using Bridge Amps and PowerLab (ADInstruments, Colorado Springs CO, USA). In the post-sepsis VNS experiment, continuous arterial pressure was recorded during VNS performed 1 h after LPS administration. Mean arterial pressure and heart rate were derived from the arterial-pressure waveform using LabChart software (ADInstruments). Representative MAP and HR traces from one LPS-treated mouse are shown in Fig. 3A and B.

### RNA extraction and quantitative real-time PCR (qPCR)

Total RNA was extracted from kidney and spleen tissues using RNAiso Plus (Takara Bio Inc., Shiga, Japan) according to the manufacturer’s instructions. Complementary DNA (cDNA) was synthesized using PrimeScript RT Master Mix (Takara Bio Inc.). Quantitative real-time PCR was performed using SYBR Green qPCR Master Mix (Thermo Fisher Scientific) on a real-time PCR system. Gene expression levels were normalized to glyceraldehyde-3-phosphate dehydrogenase (Gapdh) and calculated using the ΔΔCt method. Primer sequences are indicated in Supplementary Table.

### Statistical analysis

Data are presented as mean ± standard error of the mean (SEM). Comparisons between two groups were performed using an unpaired two-tailed Welch’s *t*-test. For comparisons among three or more groups, one-way or two-way analysis of variance (ANOVA) followed by Tukey’s post hoc test was used. A P value of < 0.05 was considered statistically significant. All statistical analyses were performed using GraphPad Prism version 10 (GraphPad Software, San Diego, CA, USA).

## Results

### Vagus nerve stimulation before sepsis onset suppresses inflammation

To evaluate the preventive effects of VNS, stimulation was performed 1 h before sepsis induction via intraperitoneal LPS injection (pre-sepsis VNS). Plasma TNF-α levels were measured 4 h after LPS administration. Preventive VNS significantly suppressed the increase in plasma TNF-α levels compared with the control group (Figs. 1A, 1B). qPCR analysis of spleen samples showed that the mean expression levels of Tnf, Il-1b, and Ccl2 were lower in the LPS-treated VNS group than in the corresponding LPS-treated sham group; however, none of these differences reached statistical significance (Tnf, p = 0.9065; Il-1b, p = 0.2730; Ccl2, p = 0.6293) (Fig. 1C).

**Fig. 1:**
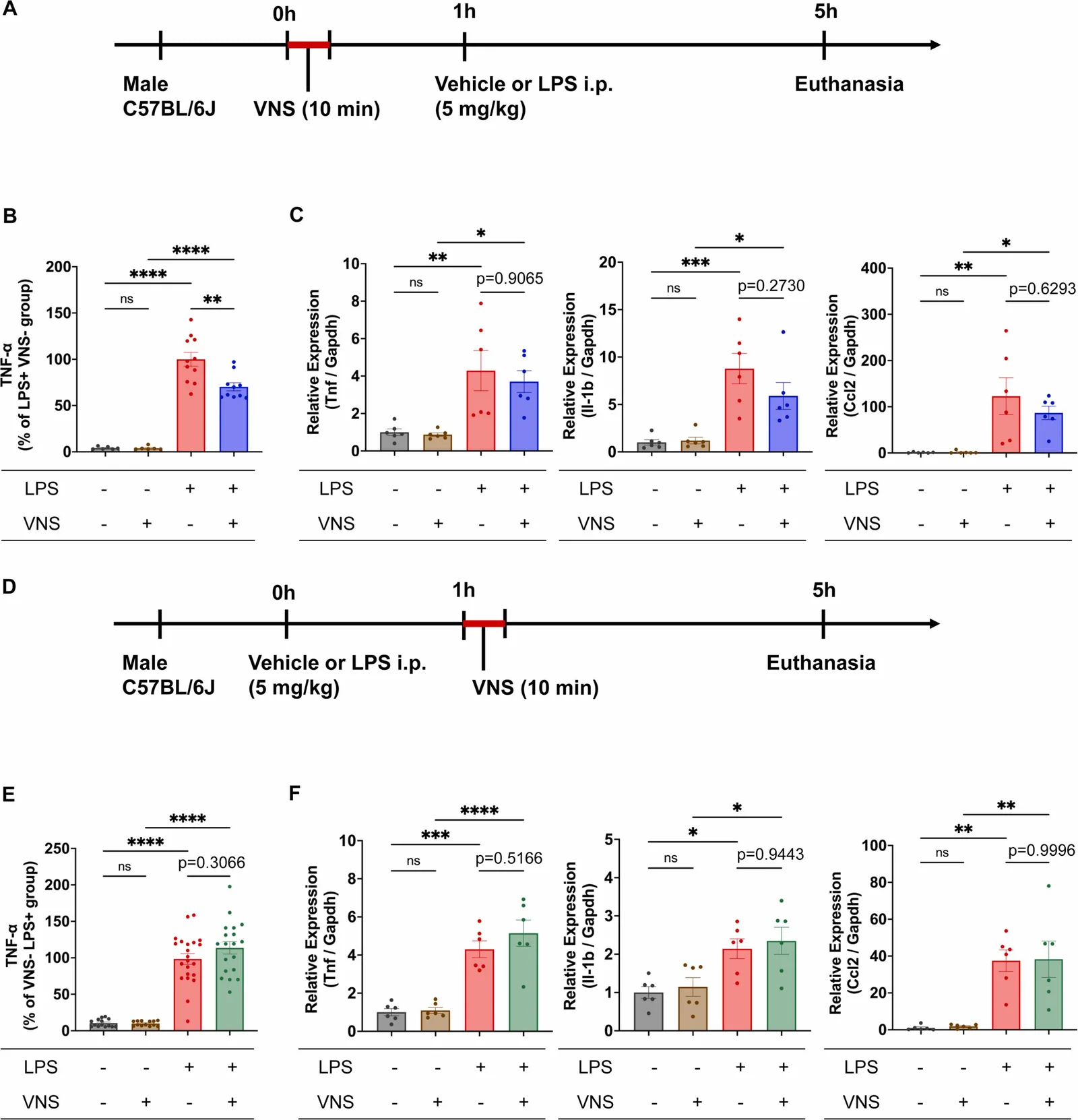
Vagus nerve stimulation suppresses inflammation before but not after sepsis onset. (A, B) Plasma TNF-α levels were significantly elevated following intraperitoneal LPS administration. Preventive vagus nerve stimulation (pre-sepsis VNS), performed 1 h before LPS injection, significantly suppressed the increase in plasma TNF-α levels compared with control mice (n = 6 for non-LPS groups; n = 10–11 for LPS groups). (C) The mean splenic expression levels of Tnf, Il-1b, and Ccl2 were lower in the LPS-treated pre-sepsis VNS group than in the corresponding LPS-treated sham group, although none of the differences reached statistical significance (Tnf, p = 0.9065; Il-1b, p = 0.2730; Ccl2, p = 0.6293) (n = 6 per group). (D, E) Therapeutic vagus nerve stimulation (post-sepsis VNS) was performed 1 h after sepsis induction. In contrast to the preventive model, post-sepsis VNS failed to suppress plasma TNF-α levels measured 5 h after LPS administration (n = 14 for non-LPS groups; n = 19–22 for LPS groups). (F) The mean splenic expression levels of Tnf, Il-1b, and Ccl2 were higher in the LPS-treated post-sepsis VNS group than in the corresponding LPS-treated sham group, although none of the differences reached statistical significance (Tnf, p = 0.5166; Il-1b, p = 0.9443; Ccl2, p = 0.9996) (n = 6 per group). Data expressed as mean ± SEM. ns; not significant, *; p < 0.05, **; p < 0.01, ***; p < 0.001, ****; p < 0.0001.

### Vagus nerve stimulation after sepsis onset fails to suppress inflammation

Next, we evaluated the therapeutic effects of VNS applied 1 h after sepsis induction (post-sepsis VNS). In sharp contrast to the preventive model, post-sepsis VNS failed to suppress plasma TNF-α levels measured 5 h after LPS administration (Figs. 1D, 1E). To further investigate the local inflammatory response in the spleen, which is a critical organ for CAP-mediated immune regulation, we performed qPCR analysis. The mean expression levels of Tnf, Il-1b, and Ccl2 were higher in the LPS-treated VNS group than in the corresponding LPS-treated sham group; however, none of these differences reached statistical significance (Tnf, p = 0.5166; Il-1b, p = 0.9443; Ccl2, p = 0.9996) (Fig. 1F).

### Post-sepsis VNS does not exacerbate organ dysfunction

We further assessed whether the application of VNS during the acute phase of sepsis influenced organ dysfunction by measuring major hematological and biochemical parameters. Although sepsis is often associated with multi-organ failure, neither LPS administration nor post-sepsis VNS significantly affected blood cell counts or clinical markers of renal (Cr, BUN) and hepatic (T-Bil) injury in this model (Fig. 2A). These results indicate that while post-sepsis VNS was ineffective in suppressing inflammation, it did not further exacerbate major organ damage. The BUN/Cr ratio was higher in the LPS-treated VNS group than in the non-LPS VNS group (Fig. 2B). Also, within the LPS-treated groups, the ratio did not differ significantly between the sham and VNS groups.

**Fig. 2:**
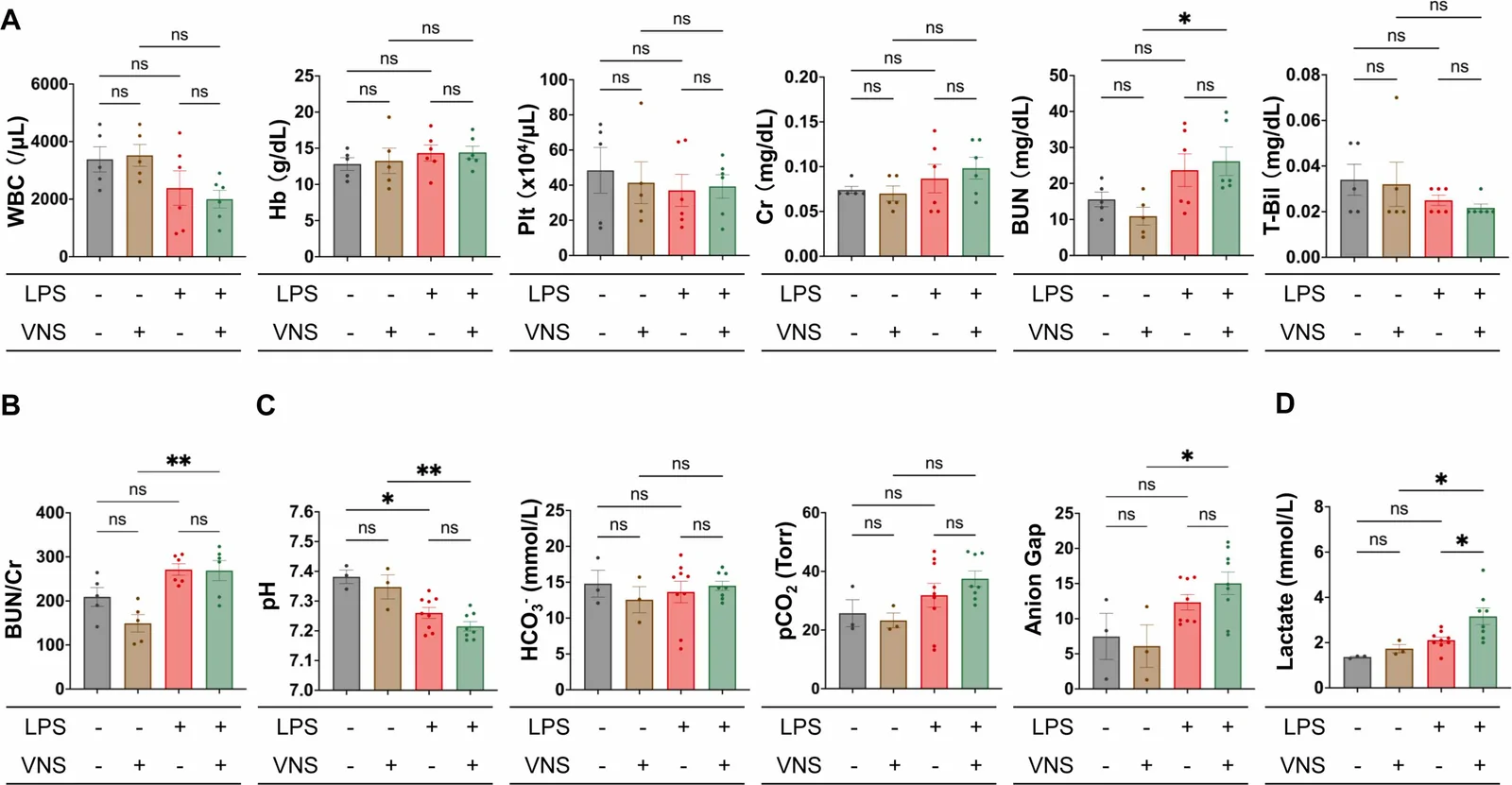
Post-sepsis VNS exacerbates lactic acidosis without worsening major organ dysfunction. (A) Comprehensive hematological and biochemical analyses were performed to assess systemic organ integrity. Blood cell counts and clinical markers of renal and hepatic injury, including creatinine and total bilirubin, were not significantly affected by either LPS administration or post-sepsis VNS (n = 5 for non-LPS groups; n = 6 for LPS groups). (B) The BUN/Cr ratio was significantly higher in the LPS-treated VNS group than in the non-LPS VNS group but did not differ significantly between the sham and VNS groups after LPS administration (n = 5 for the non-LPS groups and n = 6 for the LPS-treated groups). (C) Among VNS-treated mice, LPS administration significantly decreased blood pH and increased the anion gap compared with the corresponding non-LPS VNS group. HCO3 − and pCO2 did not differ significantly among the groups, although pCO2 was numerically higher in the LPS-treated groups. (D) Plasma lactate was significantly higher in the LPS-treated VNS group than in the non-LPS VNS group. Within the LPS-treated groups, post-sepsis VNS significantly increased plasma lactate compared with sham stimulation. The concurrent decrease in pH and increases in the anion gap and lactate were consistent with lactic acidosis in LPS-treated mice undergoing VNS (n = 3 for the non-LPS groups and n = 8–9 for the LPS-treated groups in C and D). Data expressed as mean ± SEM. ns; not significant, *; p < 0.05, **; p < 0.01.

### Post-sepsis vagus nerve stimulation exacerbates lactic acidosis

Among VNS-treated mice, LPS administration significantly increased the anion gap and decreased pH, indicating the development of high-anion-gap acidemia under septic conditions (Fig. 2C). In addition, within the LPS-treated groups, post-sepsis VNS significantly increased plasma lactate compared with sham stimulation (Fig. 2D). In an additional experiment, plasma lactate was significantly lower in mice receiving pre-sepsis VNS than in the corresponding LPS-treated sham group (Supplementary Figure S1).

These findings indicate the development of a lactate-associated high-anion-gap metabolic acidosis under septic conditions, which was further exacerbated by post-sepsis VNS. In contrast, HCO3 − levels remained low and did not differ significantly among the groups, without a further decrease in the LPS-treated VNS group. pCO2 also did not differ significantly among the groups, although it was numerically higher in the LPS-treated groups. The absence of a further decrease in HCO3 − together with the stable-to-elevated pCO2 suggests that the acid–base disturbance cannot be explained by isolated metabolic acidosis alone. Rather, the coexistence of hyperlactatemia, an increased anion gap, acidemia, and relatively elevated pCO2 suggests a mixed acid–base disorder, in which lactic metabolic acidosis is accompanied by a respiratory acidotic component.

Elevated lactate in sepsis can reflect microcirculatory alterations, impaired tissue perfusion, increased metabolic production, or reduced clearance [12]. Thus, the increased lactate following post-sepsis VNS may reflect greater metabolic stress or altered tissue perfusion under septic conditions, although tissue perfusion and lactate clearance were not directly assessed.

### Acute MAP and HR responses during post-sepsis vagus nerve stimulation

Continuous arterial-pressure recording in an LPS-treated mouse showed a transient decrease in MAP at the onset of post-sepsis VNS, accompanied by a decrease in HR, followed by partial recovery during stimulation (Fig. 3A, B). Because these traces were obtained from a single representative mouse, they describe the acute response observed in this animal but do not establish a general hemodynamic mechanism.

**Fig. 3:**
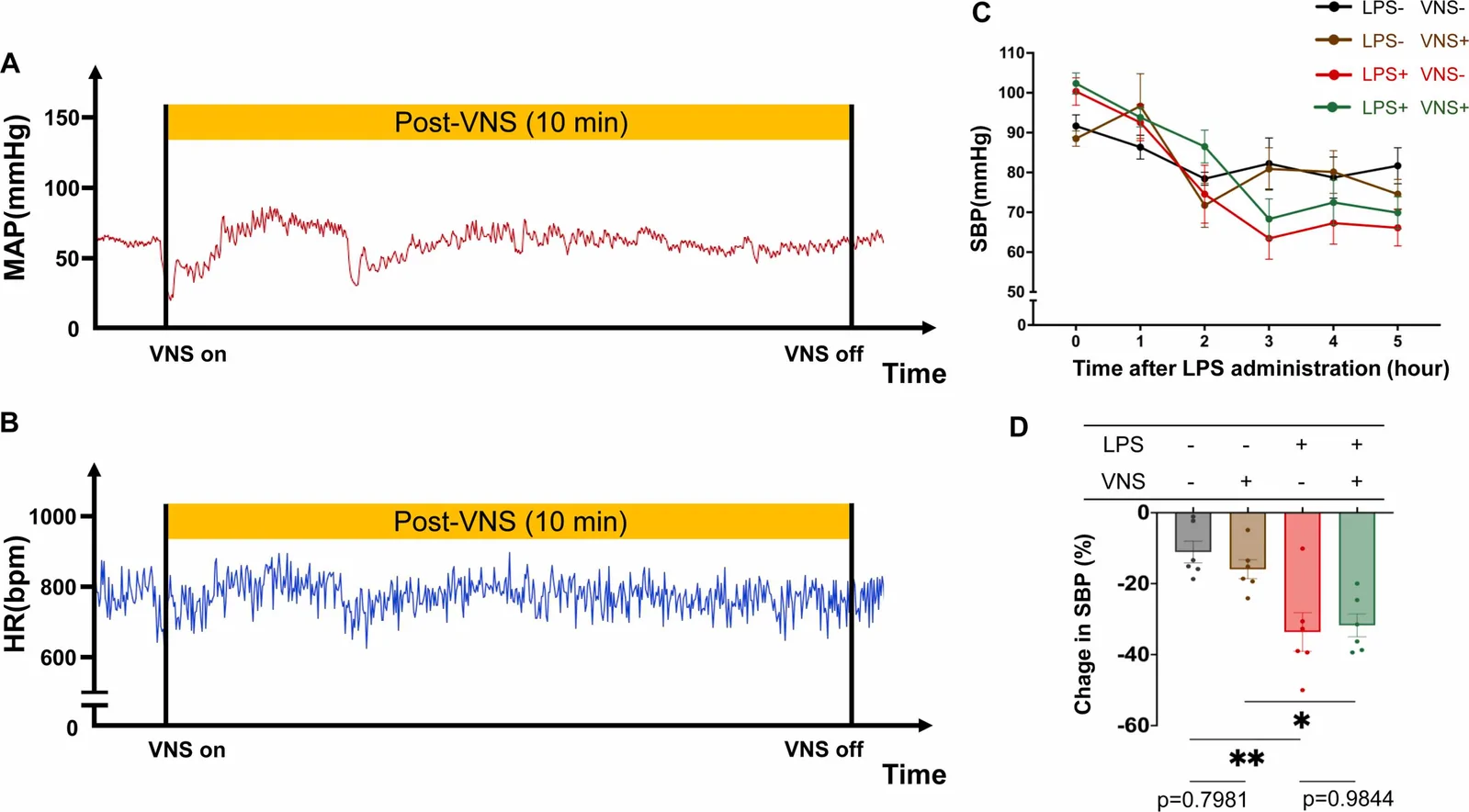
Post-sepsis VNS induces transient hypotension without worsening sustained blood pressure. (A, B) Representative continuous recordings of mean arterial pressure (MAP) and heart rate (HR) during post-sepsis VNS in an LPS-treated mouse. VNS was initiated 1 h after LPS administration and applied for 10 min. MAP and HR decreased transiently at the onset of VNS and subsequently showed partial recovery during stimulation. Representative traces from a single mouse are shown. (C) Systolic blood pressure (SBP) was measured noninvasively before LPS or vehicle administration and hourly thereafter. LPS administration resulted in a greater and more sustained decrease in SBP than vehicle administration. Within the LPS-treated groups, serial SBP did not differ significantly between the sham and VNS groups. (D) Percentage change in SBP from baseline to 5 h after LPS or vehicle administration. The reduction in SBP was significantly greater in the LPS-treated groups than in the corresponding non-LPS groups. However, no significant difference was observed between the sham and VNS groups within either the non-LPS or LPS-treated groups. Exact P values for the sham-versus-VNS comparisons are shown in the figure (n = 6 per group in C and D). Data expressed as mean ± SEM. ns; not significant, *; p < 0.05, **; p < 0.01.

### Post-sepsis vagus nerve stimulation does not worsen sustained hypotension

To further evaluate the effects of post-sepsis VNS on SBP, noninvasive blood-pressure measurements were obtained before LPS administration and hourly thereafter. Serial SBP measurements showed a graeater decrease in the LPS-treated groups than in the non-LPS groups (Fig. 3C). The percentage change in SBP from baseline to 5 h was significantly greater after LPS administration. However, no significant difference was observed between the sham and VNS groups within either the LPS-treated or non-LPS groups (Fig. 3D). These findings indicate that LPS induced sustained hypotension, whereas post-sepsis VNS did not produce a significant additional reduction in serially measured SBP.

### Fluid resuscitation restores the anti-inflammatory effect of vagus nerve stimulation after sepsis onset

Finally, we examined whether fluid resuscitation, which serves as a cornerstone of clinical sepsis management to restore tissue perfusion [13], [14], could influence the host response to VNS (Fig. 4A). When fluid resuscitation was administered 30 min after LPS injection, the anti-inflammatory effect of subsequent VNS was significantly restored, as evidenced by the clear suppression of plasma TNF-α levels (Fig. 4B). Furthermore, fluid treatment effectively attenuated the lactate accumulation that was otherwise observed following post-sepsis VNS (Fig. 4C). Serial SBP measurements in mice receiving post-sepsis VNS showed that SBP was better maintained after fluid administration than without fluid administration (Fig. 4D). At 4 h after LPS administration, SBP was significantly higher in the fluid-treated group (Fig. 4E). These findings indicate that the loss of anti-inflammatory efficacy in therapeutic VNS is linked to the impaired circulatory and metabolic context of sepsis, which can be re-established by restoring circulatory status through fluid resuscitation.

**Fig. 4:**
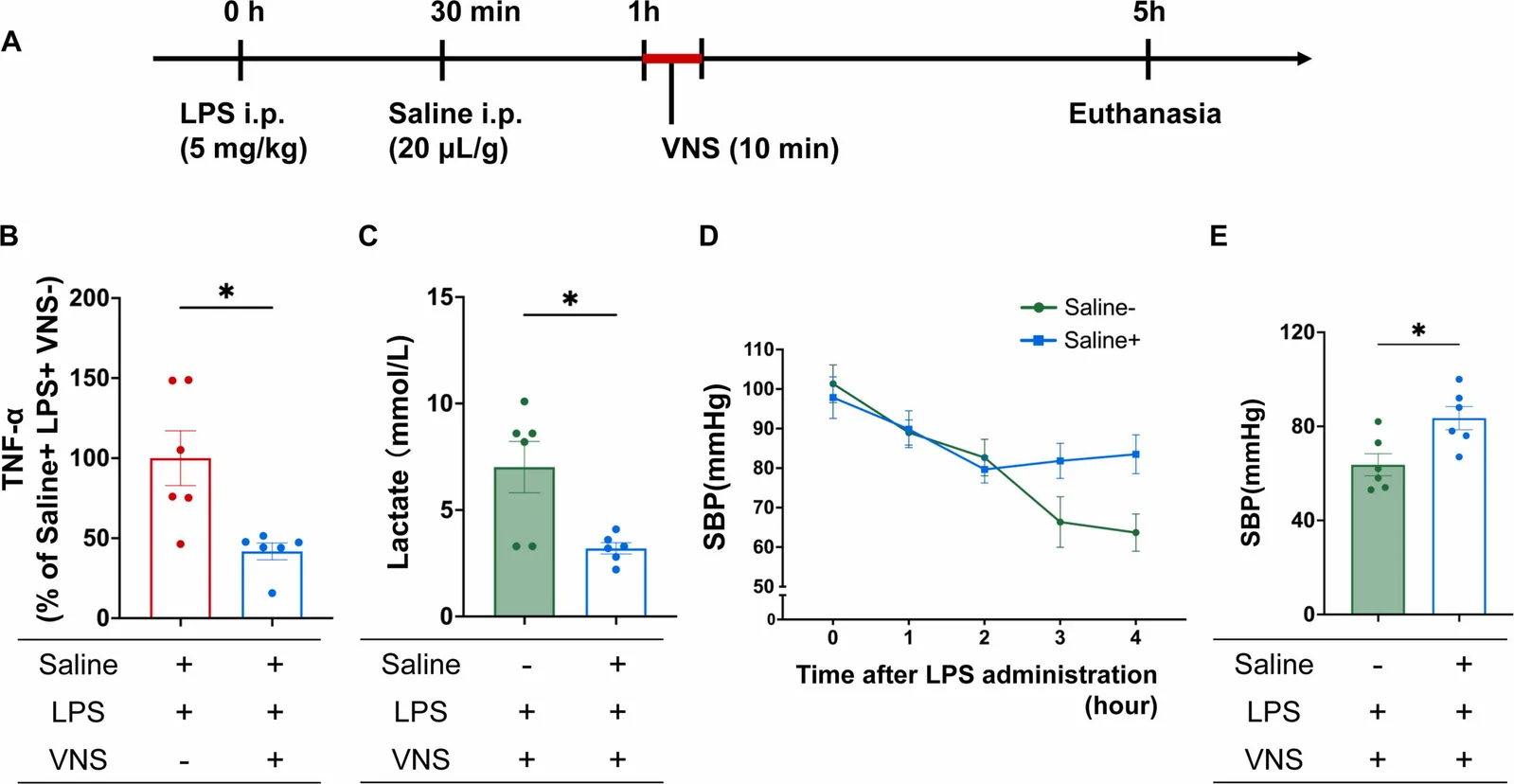
Fluid resuscitation restores the anti-inflammatory efficacy of vagus nerve stimulation after sepsis onset. (A) Experimental protocol. LPS was administered intraperitoneally at 5 mg/kg. Sterile saline was administered intraperitoneally at 20 µL/g body weight 30 min after LPS administration, followed by VNS or sham stimulation 30 min later. Mice were euthanized 5 h after LPS administration. (B) In fluid-treated mice, post-sepsis VNS significantly reduced plasma TNF-α compared with sham stimulation. (C) Among LPS-treated mice undergoing post-sepsis VNS, fluid administration significantly reduced plasma lactate compared with no fluid administration. (D) Serial noninvasive measurements of systolic blood pressure (SBP) in LPS-treated mice undergoing post-sepsis VNS with or without fluid administration. (E) SBP at 4 h after LPS administration was significantly higher in the fluid-treated group than in the non-fluid-treated group (n = 6 per group). Data expressed as mean ± SEM. *; p < 0.05.

## Discussion

In the present study, we investigated the anti-inflammatory effects of VNS before and after the onset of sepsis and evaluated the influence of circulatory and metabolic conditions on its efficacy. Preventive VNS significantly suppressed systemic inflammation, whereas VNS administered after sepsis onset failed to exert anti-inflammatory effects and was associated with elevated lactate levels. Notably, fluid resuscitation restored the anti-inflammatory efficacy of post-sepsis VNS. These findings suggest that the effects of VNS are not solely determined by the presence or absence of stimulation, but are strongly dependent on the host’s circulatory and metabolic state.

The CAP is a neuroimmune reflex that regulates systemic inflammation through vagal efferent signaling [1], [2]. In this pathway, acetylcholine-producing T cells relay neural signals to α7nAChR-expressing macrophages, leading to suppression of pro-inflammatory cytokine production [3], [4]. This anti-inflammatory signaling is mediated through inhibition of NF-κB and modulation of intracellular inflammatory pathways [15]. Previous studies have further demonstrated that transcriptional regulators such as Hes1 contribute to the downstream anti-inflammatory and tissue-protective effects of CAP activation [16]. In the present study, VNS applied under physiological conditions prior to sepsis onset significantly reduced plasma TNF-α levels, indicating that CAP activation was successfully achieved under our experimental conditions.

In addition to suppressing inflammatory cytokine production, CAP activation has also been shown to reorganize macrophage intercellular interactions and immune network behavior, suggesting that neuroimmune modulation influences the qualitative state of immune responses beyond simple cytokine suppression [17]. This concept further implies that the effects of VNS are highly context-dependent, as stimulation delivered at different stages of disease may reshape immune networks in distinct ways depending on the host physiological condition.

Previous studies have suggested that the efficacy of VNS is strongly influenced by the timing of stimulation and the stage of disease, with therapeutic responses varying considerably depending on when stimulation is delivered [18], [19]. In particular, anti-inflammatory effects appear to be more variable in acute inflammatory conditions [20], suggesting that identical stimulation may produce distinct outcomes depending on the pathological stage and physiological status of the host. Consistent with this concept, VNS applied after sepsis onset failed to suppress inflammation in our model compared with VNS before sepsis onset. Together, these findings indicate that the efficacy of VNS is context dependent and is determined not only by the stimulation itself but also by the timing of intervention and the physiological environment in which neuroimmune modulation occurs.

A key feature of CAP is its dependence on the spleen as a primary effector organ. Indeed, disruption of splenic function abolishes the anti-inflammatory effects of VNS, highlighting the importance of intact neuroimmune communication [21]. In addition, vagal signals are transmitted to the spleen via the splenic nerve, forming a critical relay in the neuroimmune circuit [22]. Because splenic immune responses rely on adequate tissue perfusion, microcirculatory alterations during sepsis may critically impair this pathway. Sepsis is characterized by profound microcirculatory dysfunction, which has been recognized as a central determinant of organ failure [12], [23]. Reduced perfusion of immune organs such as the spleen may therefore limit the capacity of vagal signals to reach immune effector cells, leading to functional impairment of CAP under septic conditions.

Another important finding of this study is the association between post-sepsis VNS and elevated lactate levels. Lactate is widely recognized as a marker of tissue hypoperfusion and metabolic stress in sepsis [13], [14], but its interpretation has evolved to include contributions from microcirculatory dysfunction and mitochondrial impairment [24], [25]. Interestingly, previous studies have reported that VNS may suppress lactate elevation; for instance, an experimental study has demonstrated that VNS attenuated hyperlactatemia and improved both hemodynamic parameters and organ function in a fluid-resuscitated porcine model of sepsis [26]. Although these findings appear to contradict our results, the discrepancy is likely explained by differences in circulatory status. In their study, VNS was applied under conditions of adequate resuscitation, whereas in our model, it was performed during circulatory instability. Under such conditions, transient VNS-induced hypotension may further impair tissue perfusion, thereby promoting anaerobic metabolism and subsequent lactate accumulation.

Notably, fluid administration restored the TNF-α-lowering effect of post-sepsis VNS and was associated with higher SBP and lower lactate levels. These findings raise the possibility that the physiological and hemodynamic state of the host influences the anti-inflammatory response to VNS. Norepinephrine is also an effective hemodynamic treatment and is recommended as the first-line vasopressor for septic shock [10], [14]. However, norepinephrine and fluid administration exert different effects on vascular tone, intravascular volume, and tissue perfusion, and whether norepinephrine can similarly restore the anti-inflammatory response to post-sepsis VNS remains unknown. Further studies directly comparing fluid and vasopressor support will be required to determine which aspects of circulatory stabilization influence CAP responsiveness. Whether circulatory support similarly modifies the effects of VNS administered before sepsis onset remains unclear and warrants further investigation in dedicated future studies.

This study used invasive cervical VNS, a conventional approach. However, other VNS modalities are available, including noninvasive transcutaneous stimulation and newer techniques such as ultrasound-based neuromodulation [6]. Because these modalities differ in neural selectivity and target engagement, they may produce different therapeutic effects in complex conditions such as sepsis. Along with timing, optimization of the stimulation modality will be important for clinical translation.

Importantly, the effects of VNS extend beyond anti-inflammatory actions and should be understood as a multifaceted physiological regulatory mechanism integrating neural, immune, endocrine, and metabolic systems [27]. Previous reports showed that sympathetic and parasympathetic signaling pathways may also cooperatively modulate macrophage inflammatory phenotypes [28], further supporting the concept that neuroimmune regulation is dynamically shaped by the host physiological environment. In addition to CAP-mediated immune modulation, VNS influences autonomic balance and regulates the hypothalamic–pituitary–adrenal (HPA) axis, thereby modulating systemic inflammatory and metabolic responses [29], [30]. These multi-axis interactions suggest that the physiological effects of VNS are highly context-dependent and may vary depending on the host’s internal state.

Taken together, our findings indicate that the efficacy of VNS is shaped not only by stimulation parameters and timing but also by the host’s physiological condition. In particular, circulatory status appears to be a critical determinant of CAP responsiveness, and our study highlights this previously underappreciated aspect.

Several limitations should be acknowledged. First, because we employed an LPS-induced sepsis model with invasive VNS, direct comparisons with studies using different sepsis models or stimulation modalities remain challenging. Second, although our findings suggest that circulatory dysfunction impairs CAP activity, we did not directly measure tissue perfusion, splenic blood flow, or neural activity. Future studies investigating microcirculation and neuroimmune interactions will be necessary to further elucidate these mechanisms.

In conclusion, our study demonstrates that the anti-inflammatory effects of VNS depend on the physiological context in which stimulation is applied. While preventive VNS effectively suppresses inflammation, therapeutic VNS after sepsis onset fails to exert anti-inflammatory effects unless circulatory function is restored. These findings support the concept that VNS efficacy represents a “context-dependent response [31],” determined by the physiological environment of the host rather than by neural stimulation alone.

## Conclusions

VNS suppresses inflammation before sepsis onset but fails to exert anti-inflammatory effects once sepsis has developed. Restoration of circulation by fluid resuscitation re-established this response, indicating that the cholinergic anti-inflammatory pathway is dependent on the host circulatory state. These findings highlight the importance of physiological context in neuromodulatory control of inflammation during sepsis.

## Author contributions

Norito Washimine, Kaori Yagishita, Sayumi Matsuo, Ryusuke Umene, Tsuyoshi Inoue designed the research studies. Norito Washimine, Kaori Yagishita, Sayumi Matsuo, Kanoko Washimine conducted the experiments, Norito Washimine analyzed the data and wrote the original manuscript, Ryusuke Umene, Chia-Hsien Wu, Yasuna Nakamura, Tsuyoshi Inoue reviewed and edited the original manuscript. Ryusuke Umene, Tomoya Nishino, Tsuyoshi Inoue supervised the study. All the authors edited and approved the final version of the manuscript.

## CRediT authorship contribution statement

**Norito Washimine:** Writing – review & editing, Writing – original draft, Visualization, Validation, Methodology, Investigation, Formal analysis, Data curation, Conceptualization. **Kaori Yagishita:** Writing – review & editing, Validation, Methodology, Investigation, Data curation, Conceptualization. **Sayumi Matsuo:** Writing – review & editing, Methodology, Investigation. **Kanoko Washimine:** Investigation. **Chia-Hsien Wu:** Writing – review & editing. **Yasuna Nakamura:** Writing – review & editing. **Ryusuke Umene:** Writing – review & editing, Supervision, Methodology, Conceptualization. **Tomoya Nishino:** Writing – review & editing, Supervision. **Tsuyoshi Inoue:** Writing – review & editing, Supervision, Resources, Project administration, Methodology, Funding acquisition, Conceptualization.

## Ethics declaration

This study was conducted in accordance with the ARRIVE (Animal Research: Reporting of In Vivo Experiments) guidelines. This study was approved by the Animal Care and Use Committee of Nagasaki University.

## Funding

This research received financial support from various sources, including the 10.13039/501100001691Japan Society for the Promotion of Science Grant-in-Aid for Scientific Research (B) (JSPS KAKENHI grant 22H03090 and 26K02431); Japan Science and Technology Agency (10.13039/501100001695JST) FOREST (Fusion Oriented Research for disruptive Science and Technology) (JPMJFR210J); 10.13039/100009666Salt Science Research Foundation (No. 22C5); SRF to TI.

## Declaration of Competing Interest

The authors declare that they have no known competing financial interests or personal relationships that could have appeared to influence the work reported in this paper.

## Data Availability

The data that support the findings of this study are available from the corresponding author, upon reasonable request.

## Appendix A Supplementary material

Supplementary Figure S1 Pre-sepsis VNS is associated with lower plasma lactate after LPS administration.VNS was performed 1 h before LPS administration, and plasma lactate was measured 4 h after LPS injection. Lactate was significantly lower in the pre-sepsis VNS group than in the corresponding sham group (n = 6 per group). Data are expressed as mean ± SEM. *P < 0.05.Supplementary material

## References

[1] Borovikova L.V., Ivanova S., Zhang M., Yang H., Botchkina G.I., Watkins L.R. (2000). Vagus nerve stimulation attenuates the systemic inflammatory response to endotoxin. Nature.

[2] Tracey K.J. (2002). The inflammatory reflex. Nature.

[3] Inoue T., Abe C., Sung S.S., Moscalu S., Jankowski J., Huang L. (2016). Vagus nerve stimulation mediates protection from kidney ischemia-reperfusion injury through α7nAChR+ splenocytes. The Journal of Clinical Investigation.

[4] Rosas-Ballina M., Olofsson P.S., Ochani M., Valdés-Ferrer S.I., Levine Y.A., Reardon C. (2011). Acetylcholine-synthesizing T cells relay neural signals in a vagus nerve circuit. Science.

[5] Koopman F.A., Chavan S.S., Miljko S., Grazio S., Sokolovic S., Schuurman P.R. (2016). Vagus nerve stimulation inhibits cytokine production and attenuates disease severity in rheumatoid arthritis. Proceedings of the National Academy of Sciences of the United States of America.

[6] Shimoyama K., Tanida M., Aruga J., Furusato T., Wu C.-H., Nakamura Y. (2026). Abdominal ultrasound activates afferent vagus nerve fibers and induces anti-inflammatory effects. Proceedings of the National Academy of Sciences of the United States of America.

[7] Uni R., Inoue T., Nakamura Y., Fukaya D., Hasegawa S., Wu C.-H. (2020). Vagus nerve stimulation even after injury ameliorates cisplatin-induced nephropathy via reducing macrophage infiltration. Scientific Reports.

[8] Matsuo S., Uni R., Wu C.-H., Nakamura Y., Umene R., Surattichaiyakul B. (2025). α7 nicotinic acetylcholine receptor agonist attenuates lipopolysaccharide-induced acute kidney injury in mice by reducing CCL2 expression in macrophages. Scientific Reports.

[9] Singer M., Deutschman C.S., Seymour C.W., Shankar-Hari M., Annane D., Bauer M. (2016). The Third International Consensus Definitions for Sepsis and Septic Shock (Sepsis-3). JAMA: The Journal of the American Medical Association.

[10] Evans L., Rhodes A., Alhazzani W., Antonelli M., Coopersmith C.M., French C. (2021). Surviving sepsis campaign: international guidelines for management of sepsis and septic shock 2021. Intensive Care Medicine.

[11] McCurdy T.R., Patrick A.L., Eltringham-Smith L.J., Bhakta V., Sheffield W.P., Fox-Robichaud A.E. (2014). Alpha-1 acid glycoprotein reduces hepatic leukocyte recruitment in murine models of either early endotoxemia or early sepsis. Microcirculation.

[12] De Backer D., Donadello K., Sakr Y., Ospina-Tascon G., Salgado D., Scolletta S. (2013). Microcirculatory alterations in patients with severe sepsis: impact of time of assessment and relationship with outcome. Critical Care Medicine.

[13] Rivers E., Nguyen B., Havstad S., Ressler J., Muzzin A., Knoblich B. (2001). Early Goal-Directed Therapy in the Treatment of Severe Sepsis and Septic Shock. The New England Journal of Medicine.

[14] Evans L., Rhodes A., Alhazzani W., Antonelli M., Coopersmith C.M., French C. (2021). Surviving Sepsis Campaign: International Guidelines for Management of Sepsis and Septic Shock 2021. Critical Care Medicine.

[15] De Jonge W.J., Ulloa L. (2007). The alpha7 nicotinic acetylcholine receptor as a pharmacological target for inflammation. British Journal of Pharmacology.

[16] Inoue T., Abe C., Kohro T., Tanaka S., Huang L., Yao J. (2019). Non-canonical cholinergic anti-inflammatory pathway-mediated activation of peritoneal macrophages induces Hes1 and blocks ischemia/reperfusion injury in the kidney. Kidney International.

[17] Nakamura Y., Matsumoto H., Wu C.-H., Fukaya D., Uni R., Hirakawa Y. (2023). Alpha 7 nicotinic acetylcholine receptors signaling boosts cell-cell interactions in macrophages effecting anti-inflammatory and organ protection. Communications Biology.

[18] Schiweck C., Sausmekat S., Zhao T., Jacobsen L., Reif A., Edwin Thanarajah S. (2024). No consistent evidence for the anti-inflammatory effect of vagus nerve stimulation in humans: A systematic review and meta-analysis. Brain, Behavior, and Immunity.

[19] Kwan H., Garzoni L., Liu H.L., Cao M., Desrochers A., Fecteau G. (2016). Vagus Nerve Stimulation for Treatment of Inflammation: Systematic Review of Animal Models and Clinical Studies. Bioelectronic Medicine.

[20] Liu F.-J., Wu J., Gong L.-J., Yang H.-S., Chen H. (2024). Non-invasive vagus nerve stimulation in anti-inflammatory therapy: mechanistic insights and future perspectives. Frontiers in Neuroscience.

[21] Huston J.M., Ochani M., Rosas-Ballina M., Liao H., Ochani K., Pavlov V.A. (2006). Splenectomy inactivates the cholinergic antiinflammatory pathway during lethal endotoxemia and polymicrobial sepsis. The Journal of Experimental Medicine.

[22] Rosas-Ballina M., Ochani M., Parrish W.R., Ochani K., Harris Y.T., Huston J.M. (2008). Splenic nerve is required for cholinergic antiinflammatory pathway control of TNF in endotoxemia. Proceedings of the National Academy of Sciences of the United States of America.

[23] Ince C. (2005). Critical Care.

[24] Hernandez G., Bellomo R., Bakker J. (2019). The ten pitfalls of lactate clearance in sepsis. Intensive Care Medicine.

[25] Singer M. (2014). The role of mitochondrial dysfunction in sepsis-induced multi-organ failure. Virulence.

[26] Kohoutova M., Horak J., Jarkovska D., Martinkova V., Tegl V., Nalos L. (2019). Vagus Nerve Stimulation Attenuates Multiple Organ Dysfunction in Resuscitated Porcine Progressive Sepsis. Critical Care Medicine.

[27] Bu, Liang Y., Hoffman BU A., Schiehser D.M., Case O., Simmons A. (2026). A Review of Vagus Nerve Stimulation for Disease: Comprehensive Theory and Evidence for Mechanisms of Action. Comprehensive Physiology.

[28] Umene R., Nakamura Y., Wu C.H., Muta K., Nishino T., Inoue T. (2023). Induction of tetraspanin 13 contributes to the synergistic anti-inflammatory effects of parasympathetic and sympathetic stimulation in macrophages. Biochemical and Biophysical Research Communications.

[29] Pavlov V.A., Tracey K.J. (2017). Neural regulation of immunity: molecular mechanisms and clinical translation. Nature Neuroscience.

[30] Bonaz B., Sinniger V., Pellissier S. (2017). Vagus nerve stimulation: a new promising therapeutic tool in inflammatory bowel disease. Journal of Internal Medicine.

[31] Mac C.-H., Nguyen G.L.T., Nguyen D.T.M., Huang S.-M., Peng H.-H., Chang Y. (2025). Noninvasive vagus nerve electrical stimulation for immune modulation in sepsis therapy. Journal of the American Chemical Society.

