# Supplementary material for: Circulatory context determines the anti-inflammatory efficacy of post-sepsis vagus nerve stimulation
Source: J Physiol Sci. 2026 Oct 15;76(3):100111. doi: 10.1016/j.jphyss.2026.100111 (PMC13718479; doi:10.1016/j.jphyss.2026.100111)
Supplement: Supplementary file 1 [file mmc2.docx]

**Supplementary Table: Real-time quantitative PCR Primer sequences**

| *Gapdh* forward | AGGTCGGTGTGAACGGATTTG |
| --- | --- |
| *Gapdh* reverse | TGTAGACCATGTAGTTGAGGTCA |
| *Tnf* forward | GCCTCTTCTCATTCCTGCTTG |
| *Tnf* reverse | CTGATGAGAGGGAGGCCATT |
| *Il1b* forward | AGCAGCATCACCTTCGCTTAG |
| *Il1b* reverse | GTGTCCAGATATTGGCATGGG |
| *Ccl2* forward | GACCTTAGGGCAGATGCAGT |
| *Ccl2* reverse | AGCTGTAGTTTTTGTCACCAAGC |
